# Review of robot-assisted partial nephrectomy in modern practice

**DOI:** 10.15586/jkcvhl.2015.23

**Published:** 2015-04-04

**Authors:** Aaron M. Potretzke, John Weaver, Brian M. Benway

**Affiliations:** Washington University School of Medicine, Division of Urologic Surgery, St. Louis, Missouri, USA

## Abstract

Partial nephrectomy (PN) is currently the standard treatment for T1 renal tumors. Minimally invasive PN offers decreased blood loss, shorter length of stay, rapid convalescence, and improved cosmesis. Due to the challenges inherent in laparoscopic partial nephrectomy, its dissemination has been stifled. Robot-assisted partial nephrectomy (RAPN) offers an intuitive platform to perform minimally invasive PN. It is one of the fastest growing robotic procedures among all surgical subspecialties. RAPN continues to improve upon the oncological and functional outcomes of renal tumor extirpative therapy. Herein, we describe the surgical technique, outcomes, and complications of RAPN.

## Introduction

Kidney cancer incidence continues to rise in the United States (U.S.) (1). The rise in incidence is at least partially attributed to the increased detection of incidental masses with more prevalent imaging (2). In 2015, an estimated 61,560 new cancer cases and 14,080 deaths will be attributed to kidney cancer in the U.S. (3). The majority of cases (> 60%) are small renal masses, < 4 cm (4).

It is well established, based on retrospective, and prospective randomized trials, that renal function after partial nephrectomy (PN) is superior when compared with radical nephrectomy (RN) (5,6). What is still unclear is whether this translates to a survival benefit, as conflicting data abounds and is debated (7–11). The most recent iteration of the American Urological Association’s guidelines references the advantages of PN and recommends it as first-line therapy for all T1a cancers, and T1b cancers in many settings (12). In accordance with this recommendation, PN utilization has increased over the past decade (13). At some centers, PN is employed for the treatment of T1a tumors nearly 90% of the time (14). In 2008, robot-assisted partial nephrectomy (RAPN) was the fastest growing robotic procedure among all surgical specialties worldwide (15). Also, Patel et al showed that over a time span corresponding to the dissemination of robotic technology (2000–2011), open RN rates decreased by 33%, PN rates increased by 15%, and RAPN rates increased to 14% at university practices and 10% at non-university practices (16).

Progression of surgical treatment has moved from open partial nephrectomy (OPN), to laparoscopic partial nephrectomy (LPN), and most recently RAPN. LPN has been deemed equally effective as OPN in terms of long-term oncological and functional outcomes (17, 18). Moreover, LPN was found to result in reduced blood loss, shorter hospital stays, superior cosmesis, and more rapid convalescence when compared to OPN. The main deterrent that has hindered the widespread adoption of LPN is the technically demanding nature of the procedure; it is therefore underutilized (19). As a result, RAPN has been studied extensively in recent years with the hope of finding a minimally invasive nephron sparing approach with a learning curve more manageable than that of LPN. RAPN appears to fit this niche as the quoted learning curve for RAPN is approximately 25 cases, whereas the learning curve for LPN is estimated to be > 200 cases (20–22). Urologists may also favor RAPN over LPN as it offers relative technical advantages (20) and decreased complication rates (23) when compared with LPN.

## Surgery

### Approach

Gettman et al. from the Mayo Clinic published the first case series of RAPN in 2004 (24). Since that time, some refinements in technique have accompanied progression of technology. There are various reports of technique in the literature which differ in minor ways (24–27). A brief description of some options is provided below.

RAPN is performed with the da Vinci surgical system (Intuitive Surgical, Sunnyvale, CA, USA). The surgery can be addressed using either a transperitoneal approach or a retroperitoneal approach. Factors that dictate which approach should be utilized include tumor location, patient’s history of prior major retroperitoneal surgery or peritoneal surgery, dense perirenal inflammation/fibrosis, musculoskeletal limitations that preclude proper positioning, and surgeon preference. The transperitoneal approach is more commonly used. This is secondary to the fact that the retroperitoneal approach is more challenging due to its confined workspace and fewer anatomic landmarks (28). However, the retroperitoneal approach does avoid bowel manipulation and allows direct exposure of the renal hilum (26).

**Figure 1.**
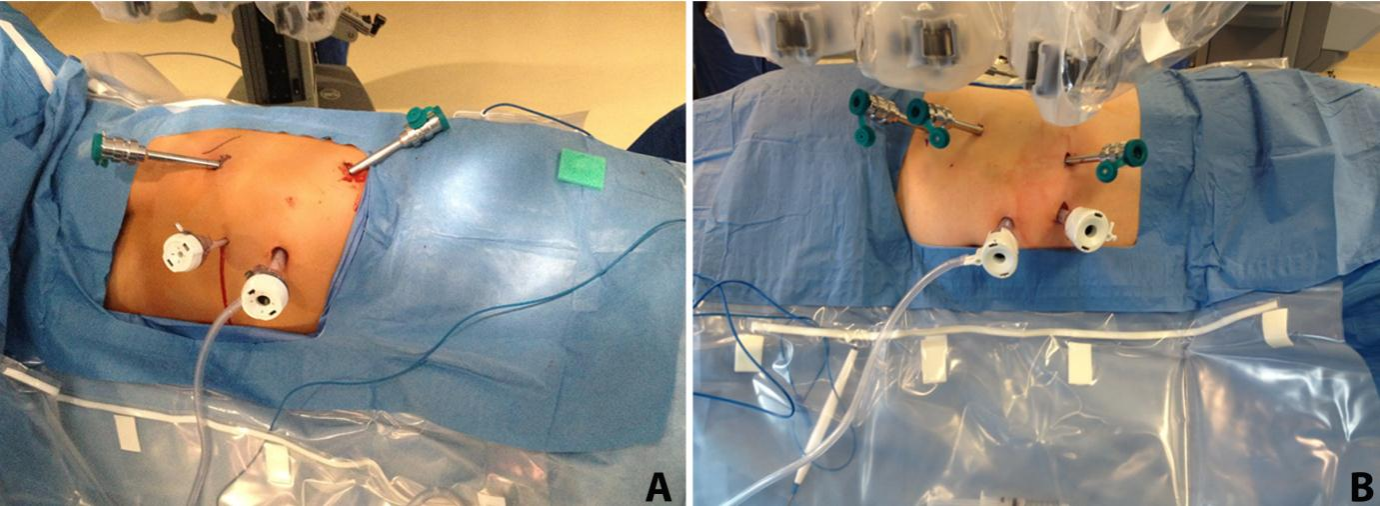
A, configuration of ports for a left RAPN with use of three robotic arms; B, configuration of ports for a right RAPN with use of four robotic arms.

Surgeon preference also dictates the number of robotic arms employed; either a three- or four-arm configuration can be used (**Figure 1**). The use of the fourth arm does provide the surgeon at the console with more control of retraction, removing some delegation to the bedside assistant.

There are multiple techniques that can be utilized in clamping the renal hilar vessels. They can be clamped individually (starting with the artery) using laparoscopic bulldog clamps or en bloc using a laparoscopic Satinsky clamp. The latter requires placement of a dedicated port. Robotic bulldog clamps provide the surgeon additional autonomy, in lieu of having to depute the assistant to the delicate task of hilar occlusion.

### Minimizing warm ischemia time

Multiple authors have demonstrated potential deleterious effects of prolonged warm ischemia time (WIT) (29–31) although its significance relative to the volume of parenchyma preserved is debated (32). Although the exact threshold is unknown, the common goal is < 30 minutes. Several novel techniques have been proposed. First, “early-unclamping” can decrease WIT. In early-unclamping, the intrarenal or hilar blood vessels are unclamped after the tumor is excised and just a preliminary repair of the deep nephrectomy bed has been performed. The parenchymal reconstruction is performed while off clamp (33). Peyronnet et al. demonstrated a decrease in WIT across 430 patients from 22.3 to 16.7 minutes (p < 0.0001). Blood loss was greater in the early unclamping group (369 vs. 240 mL, p = 0.001) (34). Next, the use of barbed suture has been proposed. Sammon et al. demonstrated a reduction in WIT from 24.7 to 18.5 minutes (p = 0.008) by using a V-Loc (Covidien, Mansfield, MA, USA) barbed suture rather than individually placed Vicryl sutures (Ethicon, Cincinnati, OH, USA). Another evolutionary technique is referred to as “zero-ischemia.” A preoperative computed tomography 3-dimensional angiogram is obtained. Induced hypotension is initiated by the anesthesiologist. The surgeon identifies and controls only the tertiary or higher-order arterial branches that feed the “tumor plus margin”, and thus, no ischemia is experienced by the renal remnant (35). Finally, “off clamp” procedures have been pursued. Tanagho et al. described a series of 29 clamped and 29 off clamp RAPN. Estimated blood loss was higher in the off clamp group (146 mL vs. 104 mL, p = 0.04), while mean change in estimated glomerular filtration rate (eGFR) was less (-4.9 vs. -11.7 mL/min, p = 0.03) (36).

### Surgical defect repair

**Figure 2.**
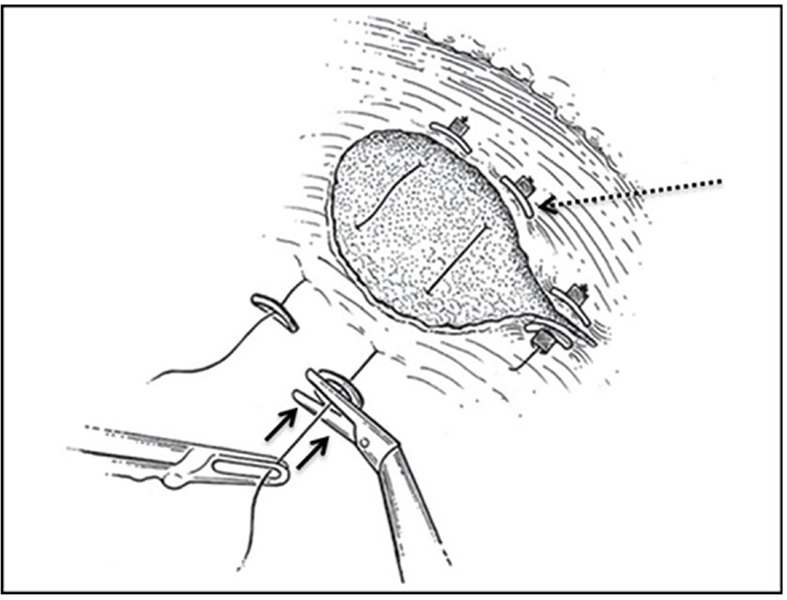
Sliding clip renorrhaphy. Solid lines indicate the direction of force applied to slide clips into position. Dashed line indicates the Hem-o-Lok clip that was placed extracorporeally on the end of the suture.

For renorrhaphy, either an absorbable monofilament or a V-Loc suture is typically used in a running fashion to repair large blood vessels and collecting system defects. A secondary layer may also be used to further approximate the deep layer of the resection bed. Next the renal capsule’s outer layer is closed with large absorbable sutures and needles. The Washington University technique of “sliding-clip renorrhaphy,” relies upon the use of Weck Hem-o-Lok clips (Teleflex, Morrisville, NC, USA), placed on Vicryl suture, on either side of the defect and then slid into place by the surgeon, to exert tension upon the repair **(Figure 2)** (25). The Hem-o-Lok clips are generally reinforced with Lapra-Ty clips (Ethicon, Cincinnati, OH, USA) to prevent backsliding of the clips. This technique is ideally suited for RAPN, as the robotic instrumentation affords the surgeon the requisite precision in dictating the degree of tension placed on the repair, effectively eliminating the need for placement of surgical bolsters in the renal defect to achieve tight closure. While other methods of renorrhaphy have been suggested, the closing tension in sliding clip renorrhaphy is superior and is relatively facile to perform (37).

### Preoperative imaging

Over the last several years, interest has grown in the development of systems to quantify and compare renal masses (e.g. PADUA, R.E.N.A.L., and C-index) (38–40). In 2009, Kutikov and Uzzo published their work on the R.E.N.A.L nephrometry score (RNS) (40). The components include: (R)adius (tumor size as diameter), (E)xophytic/endophytic properties, (N)earness to the collecting system, (A)nterior/posterior, and (L)ocation relative to the polar line. Since its inception, it was been well studied. RNS has been associated with type of surgical therapy undertaken (41,42) operative time (43) estimated blood loss (44), WIT (45), leak rate (41,46), other complications (43,47) and length of stay (48).

The benefit of preoperative assessment with tumor quantification tools is not yet entirely clear in RAPN. Some authors have found it to be related to percent functional volume preservation, nadir eGFR (49), WIT and collecting system entry (45). However, others have demonstrated either no predictive value (50), or no greater performance than a more traditional metric such as tumor size (51).

### Recent advancements

The robotic ultrasound probe (Aloka, Tokyo, Japan) can be used once the tumor is exposed in order to delineate precise tumor borders for dissection. With the da Vinci Si platform, TilePro software (Intuitive Surgical, Sunnyvale, CA, USA) can be employed for visualization of the real-time ultrasonic images. Further, this affords the surgeon greater independence from bedside assistance and obviating the need to leave the console to view images (52).

Another emerging advancement is the use of near-infrared fluorescence imaging. The component enabled in the newer da Vinci platforms is Firefly (Intuitive Surgical, Sunnyvale, CA, USA). The fluorescent marker often used is indocyanine green (ICG; Akorn, Lake Forest, IL, USA). Once a selected vessel branch is clamped, the ICG is given (5–7.5 mg), and the Firefly enacted at the console. Tissues receiving blood flow will turn fluorescent green while the ischemic tumor (and collateral tissue) will appear pale. McClintock et al. demonstrated increased renal function in the short term when compared with non-selective arterial clamping and without the use of Firefly (53).

## Outcomes

### Comparison to laparoscopic partial nephrectomy

Numerous studies now exist in the literature which favorably compare RAPN to LPN (54–60). In 2015, Choi et al. performed a meta-analysis of 23 studies, comprised of 2240 patients which compared RAPN to LPN. The authors found no difference in the following perioperative outcomes: Clavien grade 1–2 complications (p = 0.62), Clavien grade 3–5 complications (p = 0.78), change in serum creatinine (p = 0.65), operative time (p= 0.35), estimated blood loss (p=0.76), and positive surgical margins (p = 0.75). Patients undergoing RAPN had a lower rate of conversion to open (p = 0.02) or radical surgery (p = 0.0006), shorter WIT (p = 0.005), smaller change in eGFR (p= 0.03), and shorter LOS (p = 0.004) (61). No randomized trial has been done comparing the two approaches. However, given the above evidence and the inherent improvement of sewing with robotic-assistance, the robotic approach has garnered favor.

### “Trifecta”

A recently introduced concept used to evaluate PN outcomes is the “trifecta.” (62,63). The three outcomes assessed are: negative tumor margins, functional preservation, and no urologic complications. In the original paper describing the “trifecta”, Hung et al. divided patients retrospectively into four chronological eras, referred to as the discovery era (September 1999 to December 2003; n = 139), the conventional hilar-clamping era (January 2004 to December 2006; n = 213), the early-unclamping era (January 2007 to November 2008; n = 104) and the zero-ischemia era (which was performed at the authors’ institution from March 2010 to October 2011; n = 78).

Over the four eras studied, the tumors trended toward being larger (2.9, 2.8, 3.1 and 3.3 cm for the discovery, conventional hilar-clamping, early-unclamping and zero-ischemia eras, respectively; P = 0.08), but the estimated percentage of kidney function preserved was similar (89%, 90%, 90%, and 88%, respectively; P = 0.3). More recent eras were associated with increasingly complex tumors, with tumors more likely to be >4 cm in size (P = 0.03), located centrally (P < 0.009) or hilar (P < 0.0001). Nevertheless, the WITs decreased serially at 36, 32, 15 and 0 min, for the discovery, conventional hilar-clamping, early-unclamping and zero-ischemia eras, respectively (P < 0.0001). The renal function outcomes were superior in the contemporary eras, with fewer patients experiencing declines (P < 0.0001). The positive surgical margin rates were uniformly low (P = 0.7), and urological complications tended to be fewer in the more recent eras (P = 0.01). Trifecta outcomes were achieved more commonly in the recent eras and were 45%, 44%, 62%, and 68% for the discovery, conventional hilar-clamping, early-unclamping and zero-ischemia eras, respectively (P = 0.0002). In a more recent multi-institutional study, Zargar et al. reported on 1185 RAPN and 646 LPN. The authors reported a trifecta in 70% of RAPN cases, compared to 33% of LPN. WIT (18 vs. 26 min), complication rate (16.2 vs. 25.9%), and positive surgical margin (PSM; 3.2 vs. 9.7%) each favored RAPN (54). **Table 1** presents the outcomes of the largest series in RAPN.

**Table 1.** Ten largest robotic partial nephrectomy series*

Ref	N	Mean tumor size (cm)	Mean operative time (min)	Mean WIT (min)	Mean EBL (mL)	PSM (%)	Complications (%)	Mean LOS (days)	Mean f/u (months)	Mean Nephrometry
94	148	2.8	197	27.8	183	4.0	6.1	1.9	18	NR
70	183	2.9	210	23.9	132	3.8	9.8	NR	16†	NR
74	195	2.4	135†	23.8	200	1.5	NR	NR	31.1	NR
95	240	3.0†	161†	20†	100†	6.7	32.6	4†	NR	NR
96 ¥	267	2.7†	162†	17†	100†	2.4	17.6	NR	10.6†	6†
97 §	268	2.9†	205†	18†	75†	NR	22	2.8	15.4	NR
91	347	2.8†	112 (console time)	18†	100	3.6	14.7	NR	NR	PADUA score 8†
98	413	3.2	191	21	200	NA	4.3 (major)	3.6	NA	NA
99 (non-hilar vs hilar)	405 vs 41	2.6 vs 3.2†	187.4 vs 194.5	19.6 vs 26.3	208.2 vs 262.2	1.5 vs 2.4	5.4 vs 2.4	2.9 vs 2.9	NR	NR
81	886	3.0	183.6	18.8	100†	NR	139(15.6)	NR	13.3	6.9

* The most recent report of each cohort is presented.

† Median

¥ Completely endophytic tumors excluded

§ Includes only patients < 70 years old

WIT = warm ischemia time, EBL = Estimated blood loss, LOS = length of stay, PSM = positive surgical margin, f/u = follow-up, NR = not recorded, NA = not available

### Oncological outcomes

In the largest series reporting oncological outcomes to date, encompassing the work of five high-volume centers, Khalifeh et al. reviewed 943 patients who underwent RAPN. The PSM rate was 2.2%. Cases of PSM had a higher rate of recurrence and metastasis (p < 0.001). In fact, a PSM conferred an 18.4-fold higher hazard ratio for recurrence. Other authors have demonstrated similar oncologic control (55, 58, 64–66). Furthermore, in a review of modern, large RAPN series, Benway and Bhayani found that amongst >1600 patients, only seven recurrences (< 1%) were detected. The cumulative PSM rate was 2.7% (67). In comparison, PSM rates as reported by Gill et al. for LPN and OPN were 2.9 and 1.3%, respectively (17).

Limited data is available regarding long-term oncologic outcomes in RAPN given its relatively recent dissemination. In 2013, Khalifeh et al. assessed 427 patients with a mean tumor size of 3.0 ± 1.6 cm (68). Seventy patients had greater than three years of follow-up and 134 had at least two years. Overall survival was 97.0% at three years and 90.2% at five years. Cancer-specific survival was 98.9% at both three and five year follow-up. Kyllo et al. demonstrated similar outcomes in a study of 124 patients with median follow-up of 29 months (64). Three-year disease-free survival was 94.9% and cancer-specific survival was 99.1% based on Kaplan-Meier analysis. Long-term oncologic control with RAPN appears sound.

### Renal function

As mentioned above, it is known that RN is linked to increased chronic renal insufficiency (9, 10, 69). PN is intended to mitigate the unnecessary damage to a patient’s renal function that RN invokes. The first international, multi-center study of 183 patients showed no significant postoperative change in estimated glomerular filtration rate (eGFR; 82.2 vs. 79.4 mL/min/1.73 m^2^, p = 0.74) up to 26 months following RAPN (70). It should be noted that OPN is also comparable to clamped RAPN in terms of percent changes in eGFR (71). Zargar et al. assessed 99 patients with mercapto-acetyltriglycine renal scan after RAPN (72). They found the median (interquartile range) of total eGFR preservation and ipsilateral renal function (IRF) to be 83.8 (75.2 - 94.1%)% and 72.0 (60.3–81.0)%, respectively. In their cohort, volume of normal parenchyma removed, WIT > 30 minutes, body mass index, and the operated kidney’s preoperative eGFR were predictive of IRF preservation. Although the kidney on which was operated will be affected, Kumar et al. reported an interesting finding (73). It seems performing RAPN on patients with baseline chronic kidney disease (CKD) may be especially beneficial relative to other treatments. It has been shown that those with baseline CKD have a smaller magnitude of renal function decline compared to those with normal preoperative renal function.

Long-term depictions of change in renal function are developing. In a report from 2015, Kim et al. found that patients undergoing RAPN recovered more renal function in the long-term (60 months) than those who underwent LPN (74). The pattern of renal function recovery included a significant depression of renal function at ~3–9 months, and a gradual increase after reaching nadir. In the RAPN group, the nadir was 91.2% of the baseline eGFR. The renal function recovered to 95.2% of the preoperative value at 60 months.

### Tumors greater than 4 cm

As experience with RAPN has accumulated, the indications have expanded to larger tumors. Petros et al. retrospectively reviewed 445 consecutive patients from four centers; 85 patients had tumors > 4 cm (stage T1b) (75). Functional outcomes and complications were similar to those with smaller tumors, and there were no positive margins. Other series of non-robotic PN have demonstrated similar overall and cancer-specific survival for PN versus RN in T1b tumors (76,77). But, given the 2–5% lifetime incidence of contralateral renal cancer (78), it is prudent to consider PN, and RAPN, for appropriate patients.

## Complications

Early series of RAPN reported rates of complications as high as 20% (21). The complication rates in contemporary RAPN series, even those including large, complex tumors, remain similar (8.6–20.0%) (57–59). These overall complication rates are comparable to the reported complication rates of 13.7 and 18.6% in patients undergoing OPN and LPN, respectively (17). Furthermore, a study by Simhan et al. found a similar major and minor complication rate between RAPN and OPN (71). In a multi-institutional study of 450 patients who underwent RAPN, complications were stratified using the Clavien-Dindo classification system (79, 80). Seventy-one patients experienced a complication (16%), with eight intraoperative and 65 postoperative complications; 54 complications were classified as Clavien Grade I or II (12%), which required conservative management only, whereas 17 were Clavien Grade III or IV (4%) and necessitated subsequent intervention. This is comparable to another multi-institutional study of 886 consecutive cases of RAPN performed at five U.S. centers which reported an overall complication rate of 15.6%, with intraoperative and postoperative complication rates of 2.6 and 13.0%, respectively. Postoperative complications were classified as Clavien grade I–II in 77.0% of cases and grade III–IV in 23.0% (81) Updated data from this series now includes 1838 patients, an intraoperative complication rate of 2.1% and overall complication rate of 17.2%. The majority of the complications were considered Clavien 1–2 (72.5%) (82). Of all complications, hemorrhagic complications occurred in 71 (24.9%) patients, genitourinary in 72 (25.2%), pulmonary in 38 (12.4%), cardiovascular in 34 (11.1%), gastrointestinal in 26 (8.5%), infectious in 22 (7.2%) and other in 21 (6.9%) patients. Fifty-one patients (2.7%) required perioperative transfusion, 10 (0.05%) required angioembolization, and 5 (0.2%) required surgical exploration for postoperative hemorrhage. Urine leaks developed in 13 (0.7%) of patients and 10 (0.05%) patients developed postoperative acute renal failure.

### Hemorrhage

Published postoperative transfusion rates for RAPN range from 3 to 10%, which are comparable to the 5.8 and 3.4% rates for LPN and OPN, respectively (83). Furthermore, the rates of postoperative hemorrhage after minimally invasive PN are relatively low (<5%) and are similar between laparoscopic and robotic series, with a rare need for angioembolization (0.4%) (84, 85).

In one multi-institutional analysis of RAPN complications, the reported postoperative hemorrhage rate for RAPN was 5.8%, and the intraoperative hemorrhage rate was 1.0% (hemorrhage was defined as bleeding requiring blood transfusion or therapeutic intervention) (81). Many postoperative hemorrhages arise from pseudoaneurysm or arteriovenous fistula formation which may result in delayed postoperative hemorrhage, often presenting several weeks after discharge (57).

Intraoperative techniques used to decrease the risk of hemorrhage include the use of a deoxidized cellulose bolsters during renorrhaphy to provide compressive hemostasis (86); the use of a gelatin matrix thrombin sealant, which has been reported to reduce postoperative hemorrhage from 11.8% to 3.2% (87). The use of “sliding-clip” renorrhaphy, and the use of barbed V-Loc sutures (Covidien, Mansfield, MA) during reconstruction, allows the even distribution of tension across the surgical bed to control transected vessels and reduce the likelihood of postoperative bleeding (25). Although not presently validated, checklists to prepare for and manage intraoperative hemorrhage are available (88).

### Urine leak

Urine leak was formerly the most common postoperative complication of OPN with a rate of 17.4% (89), adding significant morbidity to the procedure. Minimally invasive approaches afford lower rates of urine leak when compared to open approaches (41). Reported rates of urine leak range from 0.6 – 2.5% (90). The lowest reported leak rate of 0.6% comes from a RAPN cohort of 347 patients described by Ficarra et al. (91).

## Cost

RAPN is considerably more expensive compared to LPN in the typical setting. However, in an efficient hospital and surgical system, the difference can be minimized to just $334 per case (92). Furthermore, as many studies suggest that complications are lower in RAPN compared to LPN, it may be reasonable to expect that total costs would narrow further. As health policy changes, penalties for readmissions may be assessed by the Center for Medicare and Medicaid Services. Such potential policy would make those operations with fewer readmissions due to complications more prudent (93).

## Conclusion

PN offers improved renal function and similar survival to RN. RAPN facilitates the performance of minimally invasive PN due to its short learning curve. The breadth of cases undertaken for RAPN continues to expand with enduring success. Innovation continues to make RAPN an attractive and relatively facile technology with which to provide superb care for patients with renal tumors. Future research will be directed toward refining techniques to minimize WIT and to improve upon the RAPN’s consistency in achieving the “trifecta.”
